# As We See It

**Published:** 1900-09

**Authors:** G. Alden Mills

**Affiliations:** New York


### AS WE SEE IT.

BY DR. G. ALDEN MILLS, NEW YORK.

In the May number of the *Dental Cosmos* a liberal outlay of contributions on what some see fit to characterize as the hackneyed subject of "Riggs's disease," "pyorrhœa alveolaris," etc., Professor Harlan has reviewed much on this subject, and he has gone over the ground in a fairly liberal manner, as much so as most men do who have preconceived views. This review received due attention by several present at the reading. Too many think that, because we differ, it is always due to prejudice. Too often this may be so, but putting this aside, any one who is really intelligent regarding the subject cannot fail to find much that will be helpful to a more profitable consideration. It is thought quite possible, as remarked by Dr. Rhein, that, could all those that have well-digested thought in this direction come together to confer, it would be found that there would be more agreement than would appear.

It may be found that some speakers felt that the subject of treatment did not receive due attention, simply because it was not on the programme. It was the etiology which was under discussion. We must learn to take men's views with kindness, when we know them to be forceful writers, and read between the lines for the larger thought or meaning. Dentistry is a youthful profession as yet. The faith is strong that we will be able to develop our own needs. No one so well knows as the dental scientist what is required to strengthen our ranks. We are not behind any calling of prospective men that are not yet through with investigations, and by continued conferences, the exchange of views, and a courteous demeanor, solid, practical deductions will follow.

Turning to the paper which follows Professor Harlan's,—Dr. Henry S. Nash's on "Alveolar Necrosis,"—we go over it in its published form and account it as one of the most important papers, as a whole, that has ever been given upon the subject. This paper deals precisely with a feature that has been much emphasized by those familiar with practical experience, because of the utter inability of the mass of dentists to recognize the fine pathological expressions; hence the neglect to deal with the disorder at its initial manifestations. Very few practitioners have the ability by nature or by teaching to discern a surgical condition. This fact justifies the declaration that it does not come within the domain of the dentist, but in that of the surgeon, who may be a dentist. If one has not surgical talent, he cannot be a surgeon. The late Dr. Riggs often remarked that this matter would not become intelligently considered until it was plainly seen to be a field for surgery. We give an illustration that we have given before: The doctor was corresponded with concerning a patient who was to be sent to him by a dentist holding a medical degree; he informed the doctor that it was a case of "cleaning teeth;" the doctor's reply was that there was no need to send the patient to him for such an operation.

It is our belief that Dr. Nash has stated it truly that these initial symptoms appear and disappear according to the fluctuations of systemic conditions, but sooner or later cause death of the alveolus; and there is no doubt that in some cases a sequestrum is formed. We have had two marked cases of the process being separated around bicuspids. In this field, so prolific with death of the process, is the ground for the emphasis of the value of Dr. Riggs's discoveries, and which were given to the profession. All who know what he taught, and have practised it, will recognize its true value. Many see only "tartar," and hence the wholesale neglect of these cases. They do not take them in hand with any thought that they have inability to comprehend a field of surgery which they know not how to deal with. This has long been apparent, and has often been expressed, and it is with satisfaction that one has appeared in the field who intelligently confirms the ground that the writer has so long stood upon. It has often been said in public and in private that the cause of such laxity of belief in a remediable practice was wholly because no effort was made to meet the demand that existed in this dead alveolus, because it was not recognized. Just here Dr. Riggs dealt, and by a surgical operation with the Riggs instruments, as he devised them, he removed this dead alveolus and made a simple wound, and nature came to the rescue. The letter published in the revised edition of Dr. Nash's book (prepared by the writer by his invitation) places decided emphasis upon this fact. It can be said, without fear of ever being intelligently disputed, that Dr. Nash's writings are giving us more intelligence than anything that has gone before. Nothing is hazarded in repeating what has often been said, that if practitioners did not recognize the facts pointed out, they would never contribute to the skilful management of this much-misunderstood disorder. By an intelligent discrimination in the initial phase of this alveolar disorder a vast amount of distress will be saved to our trusting clientele.

The writer desires to place himself on record with Dr. Nash concerning what he has said about Dr. Fraenkel's views that he classifies them as functional, arising from ataxic or disordered functioning of the sympathetic nervous system. It is firmly believed it will be so proved. I expressed myself decidedly but briefly regarding Dr. Talbot's late book, believing, as I did, I could see no ground for his conclusions on the low animal field that he was investigating. Sympathetic disorders in a human being lead us entirely into another realm of mental activity, which can have no bearing in the lower order of the animal kingdom. Those who indulge in evolution as dealing with mankind are led into a multiplicity of error. Man can choose his ground of belief, but some day it will be seen that he may have crossed the Creator's purposes, and this supposed light will be turned into utter darkness. This may seem a deviation and out of place, but I am of the decided belief that it is sadly needed.

